# Maternal cortisol and stress are associated with birth outcomes, but are not affected by lipid-based nutrient supplements during pregnancy: an analysis of data from a randomized controlled trial in rural Malawi

**DOI:** 10.1186/s12884-015-0793-8

**Published:** 2015-12-22

**Authors:** Christine P. Stewart, Brietta M. Oaks, Kevin D. Laugero, Ulla Ashorn, Ulla Harjunmaa, Chiza Kumwenda, David Chaima, Kenneth Maleta, Per Ashorn, Kathryn G. Dewey

**Affiliations:** 1 Program in International and Community Nutrition, Department of Nutrition, University of California, Davis, CA USA; 2 Western Human Nutrition Research Center, USDA-ARS, Davis, CA USA; 3 Department for International Health, USDA-ARS, Tampere, Finland; 4 Department of Community Health, University of Malawi College of Medicine, Blantyre, Malawi; 5 Department of Paediatrics, Tampere University Hospital, Tampere, Finland

**Keywords:** Cortisol, Stress, Preterm birth, Birth weight, Lipid-based nutrient supplements, Multiple micronutrient supplements, Malawi, Pregnancy

## Abstract

**Background:**

Prenatal micronutrient supplements have been found to increase birth weight, but mechanisms for increased growth are poorly understood. Our hypotheses were that 1) women who receive lipid-based nutrient supplements (LNS) during pregnancy would have lower mean salivary cortisol concentration at 28 wk and 36 wk gestation compared to the multiple micronutrient (MMN) and iron-folic acid (IFA) supplement groups and 2) both salivary cortisol and perceived stress during pregnancy would be associated with shorter duration of gestation and smaller size at birth.

**Methods:**

Women were enrolled in the trial in early pregnancy and randomized to receive LNS, MMN, or iron-folic acid (IFA) supplements daily throughout pregnancy. At enrollment, 28 wk and 36 wk gestation, saliva samples were collected and their cortisol concentration was measured. Self-report of perceived stress was measured using questionnaires. Gestation duration was indicated by ultrasound dating and newborn anthropometric measurements (weight, length, head circumference) provided indicators of intrauterine growth.

**Results:**

Of the 1391 women enrolled in the trial, 1372, 906 and 1049 saliva samples were collected from women at baseline, 28 wk and 36 wk, respectively. There were no significant differences in mean cortisol concentrations by intervention group at 28 wk or 36 wk gestation. Cortisol concentrations were negatively associated with duration of gestation (Baseline: β = −0.05, *p* = 0.039; 36 wk: β = −0.04, *p* = 0.037) and birth weight (28 wk: β = −0.08, *p* = 0.035; 36 wk: β = −0.11, *p* = 0.003) but not associated with length-for-age or head circumference-for-age z-scores. Perceived stress at 36 wk was significantly associated with shorter newborn LAZ (*p* = 0.001). There were no significant associations with the risk of small for gestational age, preterm birth, or low birth weight.

**Conclusions:**

Maternal salivary cortisol concentration was strongly associated with birth weight and duration of gestation in rural Malawi, but these data do not support the hypothesis that LNS provision to pregnant women would influence their salivary cortisol concentrations.

**Trial registration:**

Clinicaltrials.gov identifier NCT01239693

## Background

Intrauterine growth restriction and shortened duration of gestation are risk factors for neonatal morbidity and mortality [1] and are associated with child growth faltering in many low and middle income country (LMIC) settings [2]. An estimated 15 % of children are born low birth weight and 11 % are born preterm globally [3]. Complications from preterm birth are estimated to be responsible for 35 % of the world’s 3.1 million neonatal deaths [4] and a leading cause of under-5 mortality worldwide [5]. Risk factors for smaller size at birth or shortened duration of gestation include poor nutrition during pregnancy, infections, and maternal stress [6–8].

Interventions to improve birth outcomes in LMIC have had limited success at increasing size at birth or duration of gestation. Maternal multiple micronutrient (MMN) supplements have been found to result in a small increase in birth weight of 22 g and an increase in the duration of gestation [9, 10]. Other nutritional interventions, such as provision of calcium or zinc, have been found to result in a reduced risk of preeclampsia [11] and a modest increase in the duration of gestation [12]. Lipid-based nutrient supplements (LNS) provided during pregnancy have been evaluated for their effects on birth outcomes and early childhood growth in three studies to date, in Burkina Faso [13], Ghana [14], Malawi [15].

In impoverished communities, malnutrition is compounded by a variety of physical and psychosocial stressors that may further compromise pregnancy. Acute or chronic psychosocial experiences [16] and the biologic measure of serum or salivary cortisol concentrations [17] have been associated with shortened duration of gestation and intrauterine growth restriction. Cortisol is a regulatory hormone involved in the hypothalamic-pituitary-adrenal axis (HPA) response to stress as well as in the fetal-placental-maternal neuroendocrine system regulating the maintenance of pregnancy and the timing of parturition [18]. Cortisol and corticotropin-releasing hormone (CRH) concentrations rise exponentially over the course of pregnancy due to a positive feedback mechanism via the placenta [19]. They peak at the time of delivery and have been hypothesized to serve in regulating the ‘placental clock’, determining the timing of parturition [20]. CRH promotes fetal prostaglandin and estrogen synthesis, which lead to myometrial activation and contractility [19]. Cortisol also is involved in fetal growth and maturation. The late gestation rise in cortisol concentration is related to a shift from rapid fetal growth towards tissue maturation [21]. Elevated cortisol concentration is negatively associated with IGF-1 activity [22] and, importantly, is involved in fetal lung maturation through the production of surfactant to prepare the fetus for postnatal life [23].

Despite the physiologic explanations for a normal rise in cortisol during pregnancy, higher than expected increases in cortisol and CRH concentrations have important short- and long-term negative functional consequences on the health of the pregnancy and the offspring in later life [24, 25]. In the short term, elevated CRH is associated with placental dysfunction, including shallow trophoblast invasion and poor placental vascularization which can lead to intrauterine growth restriction and preeclampsia [26]. Elevations in perceived stress and cortisol concentrations have been related to higher concentrations of proinflammatory cytokines and lower concentrations of anti-inflammatory cytokines [27, 28]. In the longer-term, excess glucocorticoid exposure has been associated with the life-long function of the HPA axis in the offspring, and has been hypothesized to increase susceptibility to a variety of conditions including depression, hypertension, type 2 diabetes mellitus, and cognitive impairments [25, 29]. The fetus is somewhat buffered from the effects of high maternal cortisol concentrations through the activity of the 11-β-hydroxysteroid dehydrogenase (HSD)-2 enzyme in the placenta, which converts maternal cortisol to an inactive cortisone [30]. Yet this is not a perfect barrier and fetal and maternal cortisol concentrations are highly correlated suggesting that abnormally high maternal cortisol concentrations could affect the developing fetus [31]. The activity of the 11-β-HSD2 enzyme appears to be sensitive to maternal malnutrition [24, 32, 33], which would result in greater fetal exposure to active cortisol, particularly during late gestation. There is some evidence that cortisol concentrations may be elevated among undernourished individuals, including short term studies suggesting that cortisol concentration increases during energy restriction [34, 35] and that stunted children have elevated cortisol concentrations and a blunted cortisol response to stressors [36–38]. One trial among Nepali pregnant women reported that late gestation cortisol concentrations were lower among women who received multiple micronutrient supplements compared to a control [39], so it is plausible that improvements in nutrition may alter cortisol concentrations. Thus, the effects of malnutrition and elevated cortisol concentrations during pregnancy may interact deleteriously on the developing fetus, with both immediate and long-term results.

In the present study, we aimed to evaluate 1) whether maternal cortisol concentration would be affected by nutritional supplementation starting early in pregnancy and 2) whether maternal perceived stress and salivary cortisol concentration during pregnancy are associated with smaller newborn size and shorter duration of gestation. These questions were examined within the context of a three-armed, randomized controlled trial of LNS, MMN, or iron-folic acid tablets (IFA) during pregnancy in rural Malawi. Our hypotheses were that 1) women who receive LNS during pregnancy would have lower salivary cortisol concentration at 28 wk and 36 wk gestation compared to the MMN and IFA groups and 2) both salivary cortisol and perceived stress during pregnancy would be associated with shorter duration of gestation and smaller size at birth.

## Methods

To evaluate these questions, we nested a substudy within the iLiNS trial in Malawi. Details of the overall study design methods and primary outcome results have been published previously [15]. In brief, the target population included pregnant women attending antenatal care through one of four hospitals or health facilities in Mangochi District in southern Malawi. Inclusion criteria were as follows: ≤20 wk gestation confirmed by ultrasound, residence in the defined catchment area, availability during the period of the study, and signed or thumb-printed informed consent. Exclusion criteria were: age <15 years old, need for medical attention due to a chronic or severe illness, diagnosed and medically treated asthma, history of peanut allergy, history of anaphylaxis or serious allergic reaction to any substance, pregnancy complications evident at enrolment visit (moderate to severe edema, blood Hb concentration < 50 g/l, systolic blood pressure (BP) > 160 mmHg or diastolic BP > 100 mmHg), earlier participation in the trial during to a previous pregnancy or concurrent participation in any other clinical trial.

A statistician independent of the research group generated randomization codes by creating four unique lists (one for each enrollment site) in blocks of nine (3 codes for each of the 3 interventions). The codes were inserted into individual opaque envelopes and eligible participants selected one from a shuffled stack of 6 envelopes. This code determined both the participant’s group allocation as well as her identification number.

Based on their randomization code, women received one of three supplements to be consumed daily throughout pregnancy: 1) small quantity LNS; 2) MMN; or 3) IFA. The nutrient content for each of the supplements can be found in Table 1. Data collectors delivered supplements every two weeks to participants and they advised women to consume the supplements daily either after a meal (IFA or MMN groups) or mixed with a meal (LNS). Data collectors monitored adherence at each distribution visit by counting any unused supplements from participants.

**Table 1:** Nutrient content of the supplements

Nutrient	IFA	MMN	LNS
Ration (g/day)	1 tablet	1 tablet	20 g sachet
Total energy (kcal)	0	0	118
Protein (g)	0	0	2.6
Fat (g)	0	0	10
Linoleic acid (g)	0	0	4.59
α-Linolenic acid (g)	0	0	0.59
Vitamin A (μg RE)	0	800	800
Vitamin C (mg)	0	100	100
Vitamin B1 (mg)	0	2.8	2.8
Vitamin B2 (mg)	0	2.8	2.8
Niacin (mg)	0	36	36
Folic acid (μg)	400	400	400
Pantothenic acid (mg)	0	7	7
Vitamin B6 (mg)	0	3.8	3.8
Vitamin B12 (μg)	0	5.2	5.2
Vitamin D (μg)	0	10	10
Vitamin E (mg)	0	20	20
Vitamin K (μg)	0	45	45
Iron (mg)	60	20	20
Zinc (mg)	0	30	30
Copper (mg)	0	4	4
Calcium (mg)	0	0	280
Phosphorus (mg)	0	0	190
Potassium (mg)	0	0	200
Magnesium (mg)	0	0	65
Selenium (μg)	0	130	130
Iodine (μg)	0	250	250
Manganese (mg)	0	2.6	2.6

At enrollment, trained nurses confirmed pregnancies and gestational age estimates using ultrasound imagers (EDAN DUS 3 Digital Ultrasonic Diagnostic Imaging System, EDAN Instruments, Inc., Shekou, Nanshan Shenzhen, China). Study nurses were trained in ultrasound assessment by two study physicians and they conducted all measurements in duplicate. Anthropometrists measured in triplicate maternal height using a stadiometer (Harpenden stadiometer, Holtain Limited, Crosswell, Crymych, UK), weight using a flat scale (SECA 874 flat scale, Seca GmbH & Co., Hamburg, Germany), and mid-upper arm circumference (MUAC) using a non-stretchable tape (Weigh and Measure, LLC, Maryland, USA). Research nurses tested for malaria using rapid diagnostic tests (Clearview Malaria Combo, British Biocell International Ltd., Dundee, UK), haemoglobin concentration (HemoCue AB, Angelholm, Sweden), and HIV (Alere Determine HIV-1/2, Alere Medical Co., Ltd., Chiba, Japan). Positive HIV tests were repeated using another whole blood antibody test (Uni-Gold HIV, Trinity Biotech plc, Bray, Ireland).

During a follow-up home visit, trained interviewers asked mothers about demographic and socioeconomic characteristics, including questions on household food insecurity. Mothers were asked to return to the clinic for repeat visits at 32 and 36 wk gestation. A follow-up home visit was also conducted at 28 wk gestation. At enrolment, 28 wk, and 36 wk gestation, interviewers asked women about stress during the previous month using the 10-item Perceived Stress Scale [40], a tool that has been used in other low-income settings [41, 42].

The research nurses collected saliva samples during the clinic visits at baseline and 36 wk gestation and during the 28 wk home visit between 8 am and 4 pm after a 30 minute fast. Time of collection, time of waking, and time of last food or drink were recorded by the nurse. Saliva collection occurred before any other measurements or sample collection. Nurses asked each woman to place an inert polymer cylindrical swab (Salimetrics Oral Swab) under her tongue for approximately two minutes, while moving her tongue and jaw as if she were chewing to stimulate saliva. The woman removed the swab and placed it in a capped tube and then it was refrigerated or placed on ice packs. Swabs were brought to room temperature, then centrifuged for 15 min at 3,000 RPM (1500 x g) to extract saliva, which then was frozen at −20 °C. After a maximum of 2 days, samples were transferred to a −80 °C freezer for longer term storage. Samples were shipped to Davis, CA for analysis. Lab technicians measured cortisol concentrations in duplicate using an ELISA method (expanded range high sensitivity salivary cortisol kit, Salimetrics, State College, PA), which can detect cortisol concentrations ranging from 0.193 to 82.77 nmol/L (0.007-3.0 μg/dL). The intra- and inter-assay coefficient of variability is 3.5 % and 5.1 %. The mean of each duplicate measure was used for analysis.

Research nurses collected venous blood samples at baseline and 36 wk gestation using 7.5 mL trace mineral-free syringe (Sarstedt Monovette, Nh4-heparin, Sarstedt Inc., Newton, NC). Lab technicians measured zinc protoporphyrin in washed red blood cells within 30 hr of collection using a hematofluorometer (Aviv Biomedical, Lakewood, NJ). They also measured soluble transferrin receptor, c-reactive protein (CRP), and alpha-1-acid glycoprotein (AGP) by immunoturbidimetry on the Cobas Integra 400 system (F. Hoffmann-La Roche Ltd, Basel, Switzerland).

Research assistants measured infants’ weight as soon as possible after birth either at home or in the health center. Of the recorded birth weights, 89 % were measured within 48 h of delivery while the remainder were back-translated from a measurement within 14 days. They also collected early neonatal measurements, including length to the nearest 1 mm using an infantometer (Harpenden Infantometer, Holtain Limited, Crosswell, Crymych, UK), weight to the nearest 10 g using an infant scale (SECA 381 baby scale, Seca GmbH & Co., Hamburg, Germany), head circumference and arm circumference using non-stretchable tape.

Women provided written informed consent or indicated their consent to participate in the study with a thumbprint. In Malawi, individuals ≥15 y are able to provide consent themselves and so parental consent was not obtained. The institutional review boards at the College of Medicine Research and Ethics Committee (COMREC), University of Malawi and the Ethics Committee of Pirkanmaa Hospital District, Finland reviewed and approved the trial protocols at all of the hospitals and health facilities.

### Statistical analysis

Sample size estimates for the main trial were calculated to be 370 per group, based on an effect size (difference between groups divided by the pooled SD) of 0.23, assuming a two-sided α = 0.05 and β = 0.2. That would correspond to a detectable difference of 0.83 nmol/L in cortisol and a 1.2 point difference in the perceived stress score (PSS). We used standard scoring methods to calculate the PSS [40]. We checked the salivary cortisol and PSS for normality using the Shapiro-Wilk test and cortisol was log transformed. We calculated Pearson’s correlation coefficients to compare log cortisol and the PSS at each time point. We also analyzed PSS and cortisol categorically. PSS was dichotomized into high or low values using a median cut-point and cortisol concentrations were grouped into quartiles based on the distributions at each measurement point (enrolment, 28 wk, 36 wk).

We used the Household Food Insecurity Access Scale [43] to estimate food insecurity and created the scores using standard criteria. An asset index was created using principal components analysis [44] based on household ownership of a set of assets (radio, television, cell phone, bed, mattress, bednet, and bicycle), lighting source, drinking water supply, sanitation facilities, and flooring materials.

For all analyses, participants were included if they had non-missing data on either cortisol or the perceived stress score at any time-point. We compared characteristics for those with complete data vs. those who were missing data on cortisol at 28 wk gestation. We also compared baseline characteristics between women in each of the three intervention groups.

To evaluate the effect of the nutritional interventions on cortisol and PSS, we tested group-wise differences using ANOVA and ANCOVA models, using the Tukey-Kramer adjustment for multiple comparisons, and *p*-values <0.05 were considered statistically significant. We considered covariates for inclusion in the model if they were significantly (*p* < 0.1) associated with salivary cortisol. These included baseline cortisol, age, gestational age, maternal BMI and height, season, malaria infection, HIV status, hemoglobin, iron status, inflammatory markers, household food insecurity, asset index, parity (primiparous or multiparous), infant sex, site of enrollment, and maternal PSS. We included time since waking and time since last meal in all models, regardless of their association with the outcome variables. Interaction terms were created by the cross product of the intervention group and maternal age, parity, baseline BMI, and infant gender and these were evaluated in linear regression models. Interaction term *p*-values <0.1 were considered to be statistically significant.

To examine the associations between cortisol or PSS and birth outcomes (duration of gestation, weight-for-age z-score [WAZ], length-for-age z-score [LAZ], head circumference z-score [HCZ]), we used linear regression models and present standardized regression coefficients. We used Poisson regression models with robust estimation of the standard errors to estimate relative risk for dichotomous birth outcomes, including preterm birth (<37 wk gestation), low birth weight (<2.5 kg, LBW), stunting (LAZ < −2), small head circumference (HCZ < −2), and small for gestational age [45]. We considered covariates for inclusion into the models based on previous literature and tested as described above. Because cortisol and the inflammatory markers are likely related to each other, but the causal pathways are unclear, we have analyzed models both with and without adjustment for the two inflammation variables.

Missing data were considered in two ways. We first compared baseline characteristics between those with complete data and those with missing data. Secondly, we imputed missing values [46] for 28 wk and 36 wk cortisol concentrations and re-analyzed the data as a sensitivity test on the primary models. Model assumptions were also checked using standard regression diagnostics for linearity, normality, leverage, and influence. All analyses were performed using SAS 9.3 (SAS Institute, Cary, NC).

## Results

Of the total of 1391 women enrolled in the trial, 1372 women had baseline data on cortisol concentration and PSS. Baseline characteristics of women in the three intervention groups are shown in Table 2. Women’s ages ranged from 15 to 49 years and there were no differences in terms of mean age, education, or other sociodemographic factors between groups.

**Table 2:** Characteristics^a^ of pregnant women in the ILiNS-DYAD trial in rural Malawi who were included in this analysis

Characteristic	IFA	MMN	LNS	*p*-value^b^
Number of participants	413	415	418	n/a
Age, years	25 (6)	25 (6)	25 (6)	0.957
Education, completed years	3.9 (3.4)	4.0 (3.4)	4.1 (3.6)	0.647
Proportion of primiparous women	18.9 %	19.6 %	20.5 %	0.863
Gestational age at enrolment, wk	16.8 (2.1)	16.7 (2.1)	16.9 (2.2)	0.765
Weight, kg	53.8 (7.4)	54.0 (8.2)	54.0 (8.0)	0.962
Body-mass index, BMI (kg/m^2^)	22.1 (2.6)	22.2 (2.9)	22.1 (2.9)	0.908
Proportion of women with a low BMI (<18.5 kg/m^2^)	5.3 %	4.4 %	6.3 %	0.530
Proportion of anemic women (Hb < 110 g/l)	17.1 %	18.7 %	19.1 %	0.775
Proportion of women with a positive HIV test	14.8 %	9.9 %	13.1 %	0.145
Proportion with a positive malaria test (RDT)	21.2 %	22.7 %	23.7 %	0.739

^a^Mean (SD) except where noted

^b^p-value obtained from ANOVA (comparison of means) or Fisher’s exact test (comparison of proportions)

**Fig. 1:**
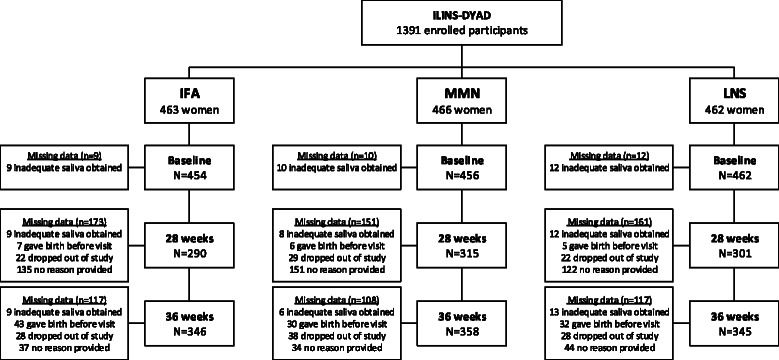
Description of sample sizes and missing data at each measurement point among pregnant women in the iLiNS-DYAD trial in Malawi. *IFA*, iron-folic acid supplement group; *MMN,* multiple micronutrient supplement group; *LNS*, lipid based nutrient supplement group

There was some loss to follow-up or missing data on cortisol or PSS at baseline, 28, and 36 wk gestation (Fig. 1), but missing data were balanced across the LNS, MMN, and IFA groups. We compared those with complete data (*n* = 883) vs. those with missing data at 28 wk (*n* = 477 women), the time point with the highest rate of missing data. We found that women with complete data were less likely to be anemic (Hb < 110 g/l; 17.0 % vs. 27.1 %; *p* < 0.001). Other characteristics, such as age, baseline cortisol, education, BMI, HIV status, prevalence of primiparity, and malaria at enrollment were generally similar between those in the analytical sample compared to those with missing data. We repeated this comparison for those with missing 36 wk data (*n* = 342) and found no significant differences compared to those with complete data.

### Differences by intervention group

Saliva samples were collected at a mean of 12:20 pm, 11:15 am, and 10:30 am at baseline, 28 wk and 36 wk, respectively. Mean cortisol concentrations at baseline differed between groups (*p* = 0.032) with the highest concentration in the IFA group (6.1 ± 4.7 nmol/L) compared to 5.4 ± 3.0 and 5.5 ± 3.3 nmol/L in the MMN and LNS groups, respectively (Table 2). Mean PSS at baseline was 14.4 (±SD: 5.6) and this did not differ by group.

**Fig. 2:**
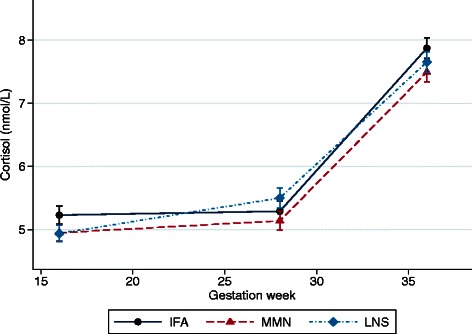
Change in salivary cortisol during gestation by intervention group. Data represent mean(SE) cortisol, adjusted for maternal age, education (completed years), socio-economic status, study site, season, baseline hemoglobin, ZPP, sTfR, time between waking and saliva collection, and time between last food or drink (besides water) and saliva collection. Measures at 28 wk and 36 wk additionally adjusted for baseline cortisol. *MMN*, multiple micronutrient; *LNS*, lipid-based nutrient supplement; *IFA*, iron-folic acid supplement

Maternal cortisol values increased during pregnancy, from 5.7 ± 3.6 nmol/L at baseline to 5.9 ± 3.3 nmol/L at 28 wk and 8.1 ± 3.2 nmol/L at 36 wk gestation (Fig. 2). PSS did not change substantially during pregnancy and there was no correlation between maternal perceived stress and salivary cortisol at any point during pregnancy (details not shown). We found no significant group-wise differences in cortisol in either crude models or those adjusted for maternal age, education, household asset index, study site, season, baseline hemoglobin, ZPP, sTfR, time between waking and saliva collection, and time between last food or drink and saliva collection (Table 3). There were also no differences in PSS between groups in either unadjusted analyses or models adjusted for maternal age, education (completed years), site of enrolment, season, and household asset index. Interactions were tested and none was found to be significant.

**Table 3:** Mean (SD) cortisol and perceived stress during pregnancy and birth outcomes by intervention group

Characteristic	IFA	MMN	LNS	*p*-value^a^
Cortisol, nmol/L				
Baseline	6.0 (4.5)	5.4 (3.2)	5.6 (3.4)	0.032
28 wk	5.7 (2.7)	5.7 (2.8)	6.2 (4.2)	0.280
36 wk	8.4 (3.5)	7.8 (2.7)	8.1 (3.3)	0.080
Perceived stress score				
Baseline	14.7 (5.6)	14.2 (5.4)	14.3 (5.4)	0.504
28 wk	14.9 (5.6)	15.2 (6.0)	14.2 (5.8)	0.079
36 wk	13.7 (5.1)	14.0 (5.4)	13.7 (4.9)	0.730

^a.^p-values represent a test of crude differences between groups using ANOVA using the Tukey-Kramer adjustment for multiple comparisons. Cortisol models at 28 wks and 36 wk were additionally tested in a model adjusting for baseline cortisol. Adjusted means did not differ from crude values and p-values were 0.14 at 28 wk and 0.24 at 36 wk

### Associations with birth outcomes

In this sample, the mean birth weight was 2971 (SD 445) g, birth length was 49.7 (SD 2.2) cm and duration of gestation was 39.4 (SD 2.1) wk. Salivary cortisol concentration was associated with a number of birth outcomes in this study population. Higher cortisol concentration at enrolment and 36 wk gestation, but not at 28 wk gestation, was associated with a shorter duration of gestation in crude and adjusted models (Table 4). For each SD increase in baseline log cortisol concentration, there was a reduction of 0.04 to 0.05 SD in duration of gestation, which translates to roughly 1 day reduced duration of gestation per SD increase in cortisol. Higher cortisol concentration at 28 wk and 36 wk gestation was significantly associated with lower mean birth weight: approximately 40 and 49 g lower per SD increase in cortisol at those respective time points. At baseline, cortisol was associated with a lower standardized birth weight in the multivariate adjusted model (*p* = 0.013), but when the model was further adjusted for CRP and AGP, the point estimate was attenuated and the difference was no longer statistically significant (*p* = 0.066). Cortisol concentration was not found to be associated with newborn WAZ, LAZ, or HCZ at any time point, however. There was less of a consistent relationship between PSS and birth outcomes. Perceived stress at 28 wk and 36 wk was not associated with duration of gestation but the 36 wk measure was significantly associated with shorter newborn LAZ (*p* = 0.001). No other associations were significant.

**Table 4:** Associations between cortisol at each time point during pregnancy with duration of pregnancy and newborn anthropometric indicators

	Crude^a^		Model 1^a,b^		Model 2^a,c^	
	β (95 % CI)	*p*-value	β (95 % CI)	*p*-value	β (95 % CI)	*p*-value
Duration of pregnancy, wk						
Baseline	−0.05 (−0.10, 0.01)	0.086	−0.05 (−0.10, −0.01)	0.015	−0.05 (−0.09, −0.002)	0.039
28 wk	−0.02 (−0.06, 0.03)	0.528	−0.02 (−0.07, 0.02)	0.305	−0.02 (−0.07, 0.02)	0.343
36 wk	−0.05 (−0.08, −0.02)	0.002	−0.04 (−0.07, −0.002)	0.032	−0.04 (−0.07, −0.002)	0.037
Birth weight, g						
Baseline	−0.08 (−0.14, −0.02)	0.007	−0.08 (−0.14, −0.02)	0.013	−0.06 (−0.12, 0.01)	0.066
28 wk	−0.05 (−0.12, 0.02)	0.189	−0.09 (−0.17, −0.02)	0.013	−0.08 (−0.16, −0.01)	0.035
36 wk	−0.10 (−0.16, −0.04)	0.0005	−0.11 (−0.18, −0.04)	0.002	−0.11 (−0.18, −0.04)	0.003
Newborn WAZ						
Baseline	−0.04 (−0.10, 0.02)	0.143	−0.04 (−0.10, 0.03)	0.265	−0.01 (−0.07, 0.05)	0.412
28 wk	−0.03 (−0.10, 0.03)	0.328	−0.06 (−0.13, 0.02)	0.129	−0.04 (−0.11, 0.03)	0.319
36 wk	−0.05 (−0.11, 0.01)	0.097	−0.05 (−0.12, 0.02)	0.174	−0.04 (−0.11, 0.03)	0.284
Newborn LAZ						
Baseline	−0.04 (−0.10, 0.02)	0.163	−0.06 (−0.12, 0.01)	0.088	−0.04 (−010, 0.03)	0.255
28 wk	−0.04 (−0.11, 0.02)	0.204	−0.06 (−0.13, 0.01)	0.111	−0.04 (−0.12, 0.03)	0.580
36 wk	−0.05 (−0.11, 0.01)	0.100	−0.02 (−0.10, 0.05)	0.545	−0.01 (−0.08, 0.06)	0.746
Newborn HCZ						
Baseline	−0.05 (−0.11, 0.01)	0.095	−0.06 (−0.12, 0.01)	0.089	−0.04 (−0.10, 0.03)	0.273
28 wk	−0.05 (−0.12, 0.02)	0.130	−0.07 (−0.14, 0.01)	0.072	−0.06 (−0.13, 0.02)	0.136
36 wk	−0.07 (−0.13, −0.01)	0.017	−0.07 (−0.14, 0.01)	0.074	−0.06 (−0.14, 0.01)	0.089

^a^Standardized regression coefficients, standard errors, and *p*-values obtained using linear regression models. Data were available for *n* = 1372, 906, and 1049 women at baseline, 28 wk, and 36 wk, respectively

^b^Adjusted for parity (primiparous or multiparous), maternal age, sex of fetus, maternal BMI, education, socio-economic score, study site, season at enrolment, gestational age at enrolment, time between waking and saliva collection, and time between last food or drink (except or water) and saliva collection

^c^Adjusted for everything in Model 1, plus AGP and CRP

*WAZ*, weight-for-age z-score; *HCZ*, head circumference-for-age z-score

We also examined the association between cortisol categorized into quartiles and dichotomous birth outcomes (Table 5). Here we found that those with cortisol in the uppermost quartile experienced a 56 % higher risk of newborn stunting compared to those in the lowest quartile (RR: 1.56; 95 % CI 1.08 to 2.25) in a multivariate adjusted model. However, when further adjusted for CRP and AGP, the effect size was attenuated (RR: 1.42; 95 % CI 0.96 to 2.09) and the difference was no longer statistically significant (*p* = 0.076). There was a relatively consistent association between higher cortisol at 28 wk or 36 wk gestation and an increased risk of LBW. Those with a cortisol concentration in Q3 or Q4 had a roughly 2-fold increased risk of LBW compared to those in the lowest quartile. Cortisol concentration was not significantly associated with SGA in adjusted models. Finally, we also examined the association between PSS dichotomized into high vs. low using a median value cutoff. High PSS at 36 wk gestation was associated with a 71 % higher risk of newborn stunting (RR: 1.71; 1.22 to 2.41; *p* = 0.002), but was not significantly associated with the other birth outcomes (data not shown).

**Table 5:** Risk of adverse birth outcomes in participants by quartile of cortisol concentrations during pregnancy

			Crude		Adjusted^a^	
	Quartile	Cases (%)	RR (95 % CI)	*p*-value^b^	RR (95 % CI)	*p*-value^b^
Preterm birth (<37 wk)						
Baseline	1	29 (8.3)	1	0.258	1	0.384
	2	34 (10.5)	1.26 (0.79 to 2.02)		1.31 (0.75 to 2.31)	
	3	35 (11.2)	1.34 (0.84 to 2.14)		1.22 (0.68 to 2.21)	
	4	30 (10.8)	1.30 (0.80 to 2.12)		1.29 (0.76 to 2.20)	
28 wk	1	20 (8.6)	1	0.753	1	0.833
	2	18 (8.9)	1.03 (0.56 to 1.90)		0.92 (0.45 to 1.87)	
	3	15 (7.0)	0.81 (0.43 to 1.54)		0.50 (0.26 to 0.99)	
	4	24 (10.0)	1.16 (0.66 to 2.05)		1.00 (0.49 to 2.04)	
36 wk	1	2 (0.7)	1	0.092	--	--
	2	9 (3.3)	4.75 (1.03 to 21.77)			
	3	6 (2.7)	3.90 (0.80 to 19.15)			
	4	8 (3.2)	4.64 (0.99 to 21.65)			
Small for gestational age						
Baseline	1	77 (28.0)	1	0.428	1	0.246
	2	81 (31.3)	1.13 (0.87 to 1.47)		1.08 (0.81 to 1.44)	
	3	83 (34.0)	1.22 (0.94 to 1.58)		1.17 (0.88 to 1.55)	
	4	68 (30.2)	1.09 (0.82 to 1.43)		1.14 (0.85 to 1.53)	
28 wk	1	53 (28.3)	1	0.5332	1	0.258
	2	37 (24.0)	0.85 (0.59 to 1.22)		0.88 (0.59 to 1.32)	
	3	58 (31.0)	1.09 (0.80 to 1.50)		1.23 (0.88 to 1.71)	
	4	56 (29.3)	1.04 (0.76 to 1.43)		1.11 (0.79 to 1.56)	
36 wk	1	61 (24.4)	1	0.028	1	0.121
	2	71 (31.4)	1.29 (0.96 to 1.72)		1.41 (1.01 to 1.98)	
	3	68 (35.4)	1.45 (1.09 to 1.94)		1.39 (0.97 to 1.97)	
	4	67 (32.8)	1.34 (1.00 to 1.80)		1.28 (0.90 to 1.81)	
Low birth weight (<2500 g)						
Baseline	1	35 (11.4)	1	0.111	1	0.170
	2	37 (12.6)	1.10 (0.72 to 1.70)		1.00 (0.64 to 1.55)	
	3	38 (13.8)	1.21 (0.79 to 1.86)		1.01 (0.63 to 1.61)	
	4	40 (15.9)	1.39 (0.91 to 2.12)		1.31 (0.85 to 2.02)	
28 wk	1	16 (7.7)	1	0.002	1	0.001
	2	15 (8.2)	1.05 (0.54 to 2.07)		1.13 (0.55 to 2.55)	
	3	30 (14.6)	1.88 (1.06 to 3.35)		2.01 (1.08 to 3.74)	
	4	35 (16.2)	2.10 (1.20 to 3.67)		2.56 (1.31 to 5.00)	
36 wk	1	15 (5.5)	1	0.003	1	0.027
	2	23 (9.1)	1.66 (0.89 to 3.11)		2.54 (1.15 to 5.59)	
	3	36 (16.7)	3.03 (1.71 to 5.39)		3.15 (1.46 to 6.78)	
	4	27 (11.7)	2.13 (1.16 to 3.90)		2.14 (1.01 to 4.56)	
Newborn stunting (HAZ < −2)						
Baseline	1	45 (15.3)	1	0.099	1	0.072
	2	42 (15.0)	0.98 (0.67 to 1.44)		1.17 (0.80 to 1.72)	
	3	41 (15.3)	1.00 (0.68 to 1.48)		1.02 (0.68 to 1.52)	
	4	48 (21.3)	1.39 (0.96 to 2.01)		1.56 (1.08 to 2.25)	
28 wk	1	25 (12.1)	1	0.095	1	0.309
	2	27 (15.1)	1.25 (0.75 to 2.07)		1.24 (0.73 to 2.10)	
	3	33 (16.8)	1.39 (0.86 to 2.25)		1.49 (0.89 to 2.51)	
	4	37 (17.9)	1.47 (0.92 to 2.36)		1.16 (0.72 to 1.87)	
36 wk	1	29 (10.9)	1	0.195	1	0.281
	2	38 (15.2)	1.38 (0.88 to 2.16)		1.69 (1.03 to 2.77)	
	3	30 (14.6)	1.34 (0.83 to 2.16)		1.47 (0.86 to 2.53)	
	4	33 (15.2)	1.39 (0.88 to 2.22)		1.29 (0.74 to 2.23)	

^a^Adjusted for parity (primiparous or multiparous), maternal age, sex of fetus, maternal BMI, education, socio-economic score, study site, season at enrolment, gestational age at enrolment, time between waking and saliva collection, and time between last food or drink (except water) and saliva collection, and gestational age at enrolment

^b^*p*-value for trend obtained from Poisson regression models

**Fig. 3:**
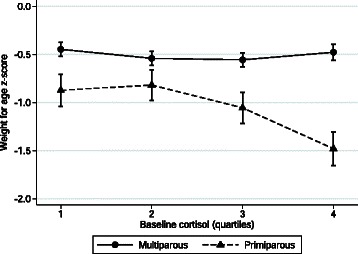
Interaction between baseline cortisol and parity on newborn weight-for-age z-score. Data represent mean (SE) weight for age z-score. *P*-value for interaction = 0.034

Interactions were tested and found to be statistically significant for parity (*p* = 0.034). Among multiparous women, there was no difference in birth weight with increasing cortisol concentration; however, among primiparous women, cortisol concentrations in the 3^rd^ or 4^th^ quartile were associated with lower mean WAZ at birth (Fig. 3). No other tested interactions were found to be statistically significant.

### Evaluation of missing data and sensitivity analyses

Using multiple imputation to estimate missing cortisol data at 28 wk and 36 wk, we re-analyzed the data and found, consistent with the primary analyses, there were no significant differences in cortisol concentration by intervention group. All coefficients in the imputed models were within 10 % of the values in the limited models (data not shown).

## Discussion

The present study tested the hypothesis that the provision of LNS would alter maternal salivary cortisol concentrations during gestation, and evaluated the associations between biologic markers of stress response or perceived stress during pregnancy and birth outcomes. Our results did not support the primary study hypothesis; we observed no association between LNS exposure and cortisol concentrations during mid- to late gestation and so we conclude that there was no effect of the intervention. In this study population, cortisol concentration at varying points during gestation was associated with duration of gestation, birth weight, and risk of LBW, but not risk of SGA. We also found that a woman’s report of perceived stress in the past month was associated with a modest increase in the risk of newborn stunting.

Our intervention effect results are similar to recent results from a trial in Ghana and two trials in Burkina Faso [47, 48], but differ from those of a study in Nepal [49]. In the Ghana study, in which women had a mean BMI of ~27 kg/m^2^ and 45 % were between 18–25 y, salivary cortisol concentrations did not differ between intervention groups overall. However, there was evidence of an interaction with maternal age whereby maternal cortisol concentrations were 1.06 nmol/L lower among younger women (<25 y) in the LNS group compared to similarly aged women in the IFA control group [50]. This difference was not observed among older women, nor was there evidence of a difference between the MMN and the IFA groups in either of the age categories. In Burkina Faso, cord blood cortisol concentrations did not differ between groups in either a trial of MMN [48] or a trial of LNS [47] during pregnancy. However, there was a significant interaction between parity and MMN supplementation, such that cord blood cortisol concentrations were higher among primiparous women in the MMN group compared to the controls (IFA), a finding not apparent among multiparous women. In the Nepal study, in which women had a mean BMI of ~19 kg/m^2^ and approximately 30 % were <19 y, maternal serum cortisol concentrations were marginally lower by 1.32 μg/dL (*p* = 0.062) in the MMN group relative to the vitamin A control group [39]. In the present study, women were similar in age (mean of 25 y) to those in the Ghana and Burkina Faso studies, but had lower mean BMI (22 kg/m^2^).

There are a number of possible explanations for why there was no impact of the supplements on maternal cortisol concentrations in this study but some apparent differences between groups in the other four trials, either in main or subgroup analyses. First, it is possible that nutrient deficiencies may not have been severe enough in our study setting to serve as a trigger for elevated maternal cortisol concentration as was seen in the Nepal trial. Maternal underweight was relatively uncommon in the Malawi study population, where only 5 % of women had low BMI (<18.5 kg/m^2^) at enrollment during early pregnancy. An alternative explanation is that the nutrients provided by LNS or MMN were insufficient to alter maternal cortisol concentrations. In this trial, there was no overall significant effect of LNS or MMN on duration of gestation or newborn anthropometric indicators [15], although the small increases in birth weight of ~50 g are in concordance with other trials of multiple micronutrients or LNS interventions during pregnancy [9, 13]. Both the Ghana and Nepal study sites reported significant increases in birth weight [14, 49]. A third potential explanation is that elevations in maternal cortisol concentration in this context were due to other non-nutritional physical or psychosocial stressors. The HPA axis is activated during infection and cortisol has important immunomodulatory effects [51], so elevated cortisol concentration may have been more strongly associated with an infectious disease burden rather than malnutrition. Cortisol response to stressors is generally blunted during pregnancy [52], which is in line with our finding of no correlation between the PSS and cortisol in this cohort.

We found that there were strong associations between maternal cortisol concentrations at some time points during pregnancy and birth weight or duration of gestation, and that the association with birth weight was strongest among primiparous women. Associations were attenuated with adjustment for CRP and AGP, which suggests that infection or inflammation were related to this relationship, either as confounding or mediating factors. There was no association with the risk of SGA, which suggests that the observed differences in birth weight were likely due to the earlier timing of birth, rather than due to intrauterine growth restriction. Late gestation perceived stress was also associated with lower newborn length. These findings are consistent with a number of other studies, including the Ghanaian and Nepali trials described above. Numerous studies have reported an association between maternal cortisol or psychosocial stress during pregnancy and adverse birth outcomes, primarily shorter duration of gestation, the descriptions of which can be found in a number of well-written reviews [16, 17]. Our findings differ somewhat from the Burkinabe trial, which measured cord blood cortisol concentrations and found there to be no association with measures of size at birth [48]. A possible explanation for this apparent difference may reflect the different pools from which the cortisol was measured and timing of measurement. We measured maternal salivary cortisol concentrations during gestation, while Roberfroid et al. measured cord serum concentrations. While maternal and fetal cortisol concentrations are correlated, they may act on fetal growth and timing of parturition through different pathways.

Strengths of the present study include the large prospective design with multiple measures during pregnancy and generally high rates of follow-up. Imputation of missing data did not substantially change the interpretation of the main findings. The randomized controlled trial design reduced the potential for confounding and bias in the analysis of the effects of the maternal supplements and cortisol concentrations during pregnancy. Unfortunately, resource constraints limited our ability to measure CRH and logistical constraints in the field limited our ability to highly control the timing and sampling protocols for salivary cortisol. Cortisol follows a strong diurnal pattern and is responsive to recent events. While we attempted to control for this by standardizing the timing of the sample collection during the course of the clinic visit, we were not able to control the timing of arrival of the participants. Thus, the samples were collected throughout the day and we adjusted for time since waking and time since last meal in all of our analyses. Ideally, one would collect multiple samples at standardized times over the course of the day and/or before and after an HPA challenge [53]; however, this was not feasible in this context. Because this measurement error was likely non-differential with regard to birth outcomes, the effect size estimates may be biased towards the null and, therefore, are a conservative estimate of the true magnitude of the association between cortisol and birth outcomes. This is supported by the consistency between our study and others on this topic. On the other hand, non-differential measurement error could have masked any true association between the supplement interventions and cortisol concentrations.

## Conclusions

We did not observe an effect of maternal supplementation with LNS or MMN on cortisol concentrations in late gestation among Malawian women. Nevertheless, we found that maternal cortisol concentrations were strongly associated with newborn anthropometric measurements and duration of gestation, reiterating findings from studies in other populations regarding the importance of this hormone for pregnancy outcomes.

## Abbreviations

CRH: Corticotropin-releasing hormone
HCZ: Head circumference-for-age z-score
HPA: Hypothalamic-pituitary-adrenal
IFA: Iron-folic acid supplements
LAZ: Length-for-age z-score
LBW: Low birth weight
LNS: Lipid-based nutrient supplements
MMN: Multiple micronutrient supplements
PSS: Perceived stress scale
SGA: Small for gestational age
TfR: Transferrin receptor
11-β-HSD2: The 11-β-hydroxysteroid dehydrogenase-2
WAZ: Weight-for-age z-score
ZPP: Zinc protoporpherin
